# Characterization of strains of *Anaplasma marginale* from clinical cases in bovine using major surface protein 1a in Uruguay

**DOI:** 10.3389/fvets.2022.990228

**Published:** 2022-09-20

**Authors:** Pablo Parodi, María T. Armúa-Fernández, Marcos Schanzembach, Daiana Mir, María José Benítez-Galeano, Nélida Rodríguez-Osorio, Rodolfo Rivero, José M. Venzal

**Affiliations:** ^1^Instituto Nacional de Investigación Agropecuaria (INIA), Plataforma de Salud Animal, Estación experimental INIA Tacuarembó, Tacuarembó, Uruguay; ^2^Laboratorio Regional Noroeste “Miguel C. Rubino”, División de Laboratorios Veterinarios “Miguel C. Rubino”, Paysandú, Uruguay; ^3^Laboratorio de Vectores y enfermedades transmitidas, Departamento de Ciencias Biológicas, CENUR Litoral Norte - Salto, Universidad de la República, Salto, Uruguay; ^4^Unidad de Genética y Bioinformática, Departamento de Ciencias Biológicas, CENUR Litoral Norte - Salto, Universidad de la República, Salto, Uruguay

**Keywords:** bovine, *Anaplasma marginale*, genotyping, MSP1a, Uruguay

## Abstract

The major surface protein 1a *(MSP1a)* gene has been used to characterize *Anaplasma marginale* genetic diversity. This pathogen causes significant productivity and economic losses to the cattle industry. The objective of the present study was to report the first characterization of *A. marginale* genetic diversity in Uruguay based on MSP1a genotypes and their putative relationship with *Rhipicephalus microplus*. This cross-sectional study was conducted between 2016 and 2020. The study included whole blood samples from clinical cases of bovine anaplasmosis obtained from 30 outbreaks located in six Uruguay territorial departments. Diagnosis was performed using Giemsa-stained smears and confirmed by nested Polymerase Chance Reaction (nPCR) targeting the *A. marginale* major surface protein *5* gene. The genetic diversity of *A. marginale* strains was characterized by analyzing the microsatellite and tandem repeats of MSP1a. Based on the microsatellite structure, four genotypes were identified. Genotype E was the most prevalent. Analysis of MSP1a tandem repeats showed 28 different strains from the combination of 31 repeats, with τ-10-15 and α-β-β-β-Γ being the most common. Repeats Γ, β, α, and γ were associated with the absence of *R. microplus* with statistical significance (*p* < 0.05). Molecular observations showed that 46.7% of the strains identified in our samples lacked the ability to bind to tick cells; therefore, they were probably transmitted by other vectors. Strain genetic diversity provides valuable information for understanding the epidemiological behavior of A. *marginale* and could contribute to the development of effective vaccines for the control of this disease.

## Introduction

Bovine anaplasmosis is caused by an obligate intraerythrocytic Anaplasmataceae agents, *Anaplasma marginale* (1), found within membrane-bound vacuoles (1 μm in size) in the cytoplasm of host cells. This bacterium belongs to the order Rickettsiales and family Anaplasmataceae (2). Bovine anaplasmosis is widely distributed throughout the world, particularly in tropical and subtropical regions (3). It is considered a major economic and production problem for cattle in enzootic tick-infested areas (4).

Different species of *Dermacentor* and *Rhipicephalus* can biologically transmit *A. marginale*. However, some *A. marginale* strains are not infective or transmissible by ticks (5, 6). In Uruguay, the only tick species related to the transmission of *A. marginale* to cattle was *Rhipicephalus microplus* (7).

*Anaplasma marginale* is often transmitted mechanically to susceptible cattle by blood-contaminated mouthparts of the bloodsucking diptera of the *Tabanus* and *Stomoxys* genera, or *via* fomites (8). In cattle, the only site for the replication is within the erythrocytes, where it develops membrane-bound vacuoles which contain−4–8 *A. marginale* (9).

The prepatent period ranges from 7 to 60 days (depending on the infective dose), and as many as 70% or more of the erythrocytes may become infected during acute infections and/or during the manifestation of clinical sings (3). The animals most susceptible to developing clinical diseases are bovines older than 1 year. Severe anemia, icterus (without haemoglobinuria), fever, weight loss, lethargy, depression, and abortion were the main clinical signs observed (10). The major postmortem findings include severe haemolytic anemia, icterus, splenomegaly, hepatomegaly, and petechial hemorrhage on the serosa surface over the heart and pericardium. All tissues were pale and blood was thin and watery (11). Cattle that survive acute infections may remain carriers for life (1).

Currently, there are different strains of *A. marginale* worldwide, with diverse epidemiological behaviors, virulence, pathogenicity, adaptation to ecological niches and induction of the host‘s immune response (12). The major surface proteins (MSP1a, MSP 4, and MSP 5) have been used for the molecular characterization of strains (5), as these are single genes that do not vary antigenically within isolates (3). In particular, the analysis of MSP1a gene sequences provides information regarding genetic diversity, evolution of host-pathogen and vector-pathogen relationships, and transmissibility of phenotypes. Moreover, these sequences can be used to compare strains in a given region (12, 13). Furthermore, using information from the MSP1a tandem repeat, it is possible to design peptide-based vaccines (12).

Therefore, MSP1a is a stable marker of strain and has been widely used to identify different strains of *A. marginale* based on N-terminal variable tandem repeats (more than 300 strains characterized by tandem repeat structures) and 5′- untranslated region (UTR) microsatellite (described eleven genotypes identified by letters A to L) located in the MSP1a gene (14–16).

There are many studies around the world that report the genetic diversity of *A. marginale*. This diversity is higher in region where the vector *R. microplus* is present (14, 15, 17, 18). This situation of wide diversity is frequently reported in South American countries where they have endemic region of *R. microplus*, both in beef and dairy cattle production (19–22).

Currently, no studies have been carried out in Uruguay to characterize the strains of *A. marginale*. Therefore, the aim of the present cross-sectional study was to characterize the genetic diversity of *A. marginale* in clinically sick animals in Uruguay and its possible relationship with *R. microplus*.

## Materials and methods

### Study design, geographical area, and sample collection

**Figure 1:**
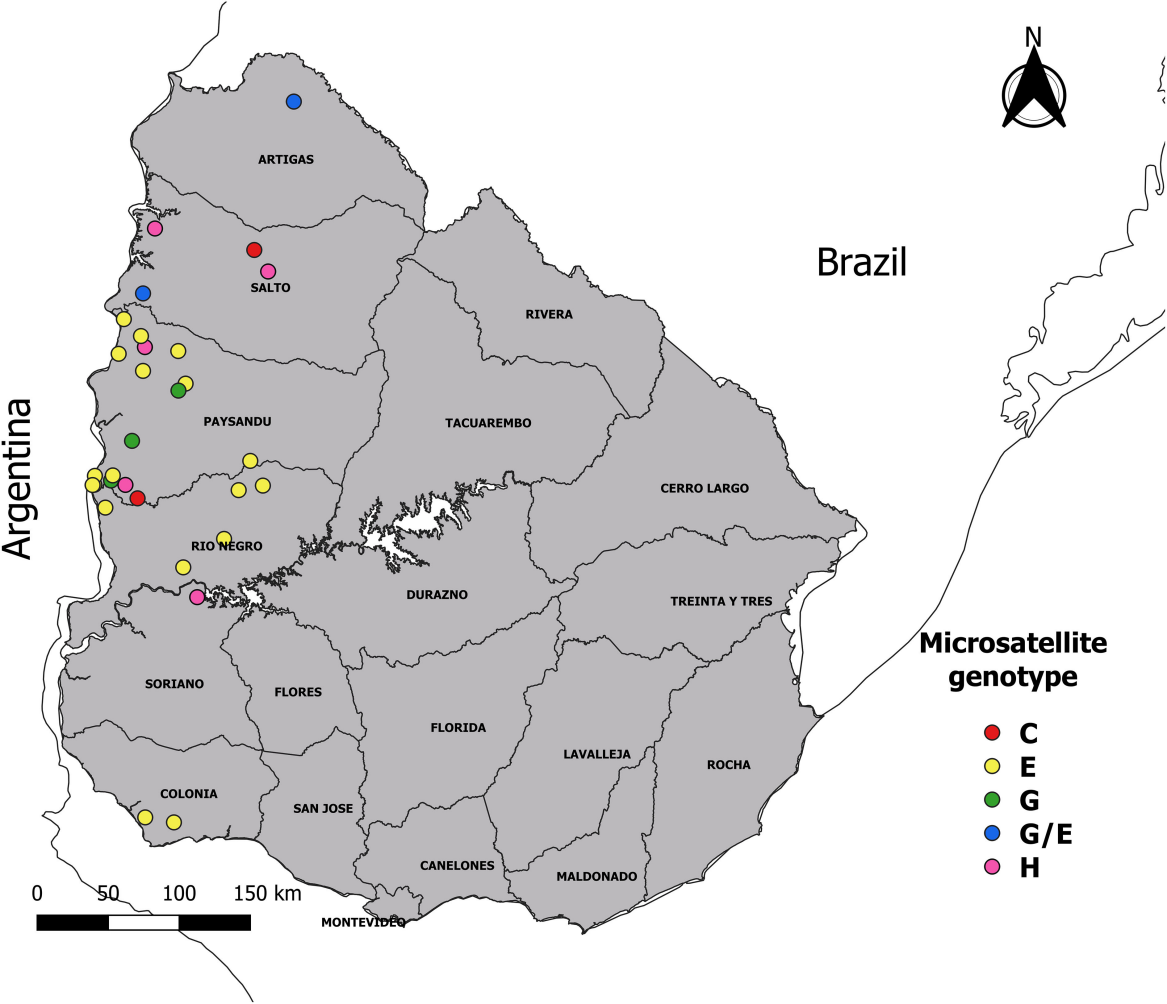
Map showing geographic location of the samples. The 60 samples of infected animals with *Anaplasma marginale* were obtained from 30 outbreaks of bovine anaplasmosis (dots with different color according to microsatellite genotype), distributed in six departments of Uruguay.

A cross-sectional study was carried out in Uruguay, a country located in the Southern Hemisphere temperate zone between parallels 30° and 35° of the South latitude and meridians 53° and 58° of the West longitude. The region has subtropical climatic conditions, with an average annual temperature of 17.5°C and an annual average rainfall of 1,200 mm. Convenience sampling was performed between August 2016 and April 2020. Sixty blood samples from tail vein in tube with potassium EDTA_3_ K anticoagulant (ethylenediaminetetraacetic acid) from clinical cases of anaplasmosis were collected from animals of different ages (cows, calves, steers, and heifers). The samples were obtained from 30 farms (outbreaks) located in six of 19 Uruguayan departments: Artigas, Salto, Paysandú, Rio Negro, Soriano, and Colonia (Figure 1). Of these 30 farms, belonged: 25 beef production cross breed herd (48 samples) and five herd of dairy cattle Holland (12 samples). These samples were sent by veterinary practitioners to the laboratory of the “División Laboratorios Veterinarios” (DILAVE) Northwest region of the “Ministerio de Ganadería, Agricultura y Pesca” (MGAP), Uruguay for diagnosis. The presence or absence of *R. microplus* has been reported for each outbreak.

Blood samples were collected from animals with fever (>39.9°C), anemia (anemia was considered when the microhematocrit was <26%; for this microtube with blood was centrifuged at 11,800 × *g* for 5 min), weakness, jaundice, and pale mucosa. The average morbidity and mortality (minimum–maximum) registered in the farms where samples were collected were 5.4% (0.2%−32%) and 2.8% (0%−25.7%), respectively.

### Blood smear and molecular diagnosis

To confirm clinical cases of anaplasmosis, smears and molecular detection using nested PCR (nPCR) were performed. For this, blood extracted from tail vein was used. Blood smears were fixed with methanol for 5 min and stained with Giemsa for 60 min. All smear readings were performed by the same trained technician. For a positive result, at least 5% of the parasitised erythrocytes had to be observed in 100 fields. The smears were viewed under a light microscope at 1,000× magnification. For molecular diagnosis, DNA extraction from 200 μl aliquots of blood was performed using the PureLink Genomic DNA Mini Kit (Invitrogen, USA) following the manufacturer's instructions. DNA was quantified using a NanoDrop 2000 spectrophotometre (Thermo Scientific) and stored at −20°C until further analysis. Molecular detection of *A. marginale* was carried out by nPCR targeting a 458 bp fragment of the MSP-5 gene, as previously described (23). In each run of the assay, a negative control (ultrapure water) and a positive control (*A. marginale* Paysandú isolate), previously sequenced and stored, were included.

### PCR targeting MSP1a gene and sequence processing

Molecularly confirmed *A. marginale* samples were used to amplify a fragment of the MSP1a gene. For this purpose, a semi-nested PCR method described by Lew et al. (24) was carried out. The primers used were as follows: 1733F (5′TGTGCTTATGGCAGACATTTCC3′) and 2957R 5′AAACCTTGTAGCCCCAACTTATCC 3′) in the first step, and 1733F and 3134R (5′ TCACGGTCAAAACCTTTGCTTACC 3′) in the second round, only when the first step showed no detectable bands. Reactions were performed in a final 25 μl volume consisting of 12.5 μl of MangoMix (Bioline; Meridian Bioscience), 6.5 μl of ultrapure water, 4 μl of sample DNA solution, and 1 μl (10 pmol) of each primer. The amplification cycling following an initial denaturation at 94°C for 3 min, 30 cycles of 30 s at 94°C, 1 min at 55°C and 2 min at 72°C, followed by a final cycle with a 7 min extension step at 72°C. The amplification protocol was performed as described by Lew et al. (24). The PCR products from each reaction were run on a 1.5% agarose gel stained with GoodView nucleic acid stain (Beijing SBS Genetech) and visualized under a UV transilluminator.

Amplicons of the expected size were purified using a PureLink quick PCR purification kit (Invitrogen) and sequenced (Macrogen, Seoul, South Korea). Sequence identities were confirmed using BLASTN (http://www.ncbi.nlm.nih.gov/BLAST). The raw sequences were assembled with the software MEGA X (25). Each sequence was carefully checked, and manual corrections were performed when necessary.

### Molecular characterization of the *A. marginale*

*Anaplasma marginale* strains were classified using microsatellite genotyping and tandem repeat (TR) composition (15, 26). A microsatellite is located in the 5′-UTR of the MSP1a gene between the putative Shine-Delgarno (GTAGG) sequence and the translation initiation codon (ATG), SD-ATG (5), containing the sequence structure GTAGG (G/ATTT)m (GT)*n* T ATG. The Shine-Delgarno and initial codon distances were calculated as (4 × *m*) + (2 × *n*) + 1, and the resulting genotypes were identified by the letters A–L (14, 16).

MSP 1a sequences were classified by TR sequence and number following the nomenclature described by de la Fuente et al. (26) and other authors (15, 16, 27). The TRs present in each sequence were classified using RepeatAnalyzer software (28). The amino acid composition of the MSP 1a repeats is described for each sample.

### Phylogenetic analysis

Phylogenetic analyses were conducted using the MSP1a amino acid sequences. Theoretical translation of nucleotide sequences into amino acid sequences was performed using the ExPASy translation tool web server (https://web.expasy.org/translate/).

A set of reference sequences for phylogenetic analysis was obtained by a BLAST search of each Uruguayan sequence against the GenBank database. BLAST's top five hits by sequence (*E*-values < 1.0 × 10–5, >700 bp long, and with available information of country and collection date) were retrieved. The repeated sequences in the dataset were removed. This resulted in a final dataset of 114 sequences from nine countries. The amino acid sequences obtained in this study were aligned with sequences retrieved from GenBank using MAFFT v7.467 program (29) and subjected to maximum likelihood (ML) phylogenetic analysis. The ML tree was inferred with IQ-TREE 1.6.1 software (30) under the JTT+F+I+G4 amino acid replacement model selected by ModelFinder application. Branch support was assessed using the approximate likelihood-ratio test based on a Shimodaira-Hasegawa-like procedure (SH-aLRT) with 1,000 replicates (31).

### Statistical analysis

The association between the TRs identified in the sample and the presence or absence of *R. microplus* was evaluated using the Phi correlation coefficient (ϕ) and Yates Chi^2^ test (X^2^Yates), with a 95% confidence level. The test was performed with the statistical software Statistical Package for the Social Sciences (SPSS), v. 28.0.0.0 (International Business Machines –IBM, USA).

## Results

All 60 samples were smear-positive, showing more than 15% parasitised erythrocytes. In addition, all samples were molecularly confirmed as *A. marginale* by MSP five nPCR. Regarding animal age, the positivity distribution was 45 cows, 13 steers, one heifer, and one calf. Sixteen outbreaks were tick-free (35 animals) and 14 outbreaks had ticks (25 animals). All animals showed clinical signs of haemolytic disease.

### Molecular characterization of *A. marginale* strains using MSP 1a

MSP1a sequence analysis showed amplicons between 670 and 1,110 bp. Based on the structure of the *MSP1a* microsatellite of *A. marginale, four* genotypes were identified. The most frequent genotype was E (45/60), followed by G (8/60), H (6/60), and C (1/60).

Tandem repeats analysis revealed 28 different genotypes were found in our 60 samples, contained between two and eight TRs.

The strains commonly observed had three (42%), five (28%), and four (10%) tandem repeats. Thirty-one TRs were found, being the most frequently identified 15, τ, 10, Γ, F, β, and α (Table 1 and Supplementary Table SI).

**Table 1:** Sequence of MSP1a tandem repeats found in the *Anaplasma marginale* strains of this study, ordered from highest to lowest presence.

**Repeat**	**Sequence of *msp1*α tandem repeat**																															**Tick**
A (ref)	D	D	S	S	S	A	S	G	Q	Q	Q	E	S	S	V	S	S	Q	S	E	–	A	S	T	S	S	Q	L	G	*	*	–
15	A	*	*	*	*	*	*	*	*	*	*	*	*	G	*	L	*	*	*	G	Q	*	*	*	*	*	*	*	*	*	*	TA
τ	T	*	*	*	*	*	*	*	*	*	*	*	*	*	*	L	*	P	*	G	Q	*	*	*	*	*	*	*	*	*		TP
10	A	*	*	*	*	*	*	*	*	*	*	*	*	*	*	L	*	P	*	G	Q	*	*	*	*	*	*	*	*	*	*	TP
Γ	T	*	*	*	*	*	*	*	*	*	*	*	*	*	*	*	*	*	*	D	*	*	*	*	*	*	*	*	*	*		TA^1^
F	T	*	*	*	*	*	*	*	*	*	*	*	*	*	*	*	*	*	*	G	Q	*	*	*	*	*	*	*	*	*		TA
β	T	*	*	*	*	*	G	D	*	*	*	G	*	G	*	*	*	*	*	G	Q	*	*	*	*	*	*	*	*			TA^1^
α	A	*	*	*	*	*	*	*	–	–	–	–	–	–	*	L	*	*	*	G	Q	*	*	*	*	*	*	*	*	*		TA^1^
3	A	*	*	*	*	*	*	*	*	*	*	*	*	*	*	L	*	*	*	G	Q	*	*	*	*	*	*	*	*	*	*	TA
ru6	T	*	*	*	*	*	*	*	*	*	*	*	*	G	*	*	*	*	*	*	A	S	T	S	*	Q	L	G				TP
γ	T	*	*	*	*	*	*	*	*	*	*	*	*	*	*	*	*	*	*	D	*	*	*	*	*	*	–	Q	L	G	*	TA^1^
B	A	*	*	*	*	*	G	*	*	*	*	*	*	*	*	*	*	*	*	D	Q	*	*	*	*	*	*	*	*	*		TP
E	A	*	*	*	*	*	*	*	*	*	*	*	*	*	*	*	*	*	*	*	*	*	*	*	*	*	*	*	*	*	*	TP
M	A	*	*	*	*	*	*	*	*	*	*	*	*	*	*	*	*	*	*	G	Q	*	*	*	*	*	–	Q	L	G	*	TP
5	A	*	*	*	*	*	*	*	*	*	*	*	*	*	*	*	*	*	*	D	*	*	*	*	*	*	*	*	*	*	*	TA
9	A	*	*	*	*	*	*	*	*	*	*	*	*	*	*	*	*	*	*	D	*	*	*	*	*	*	*	S	*	*	*	TA
4	T	*	*	*	*	*	*	*	*	*	*	*	*	*	*	L	*	*	*	G	Q	*	*	*	*	*	*	*	*	*	*	TA
EV7	T	*	*	*	*	*	*	*	*	*	*	*	*	*	*	L	*	P	*	G	Q	*	*	*	*	*	*	*	*	V	G	TA
T	A	G	*	*	*	*	G	*	*	*	*	*	*	*	*	*	*	*	*	D	Q	*	*	*	*	*	*	*	*	*	*	TP
13	T	*	*	*	*	*	*	*	*	*	*	*	*	*	*	L	*	*	*	D	Q	*	*	*	*	*	*	*	*			TP
100	T	*	*	*	*	*	*	*	*	*	*	*	*	G	*	L	*	*	*	G	Q	*	*	*	*	*	–	Q	L	G	*	TP
154	A	*	*	*	*	*	*	*	*	*	*	*	*	*	*	L	*	*	*	D	Q	*	*	*	*	*	–	Q	S	G	*	TP
C	A	*	*	*	*	*	G	*	*	*	*	*	*	*	*	*	*	*	*	G	Q	*	*	*	*	*	*	*	*	*		TP
m	A	*	*	*	*	*	*	*	*	*	*	*	*	*	*	*	*	*	*	G	Q	*	*	*	*	*	*	S	*	*	*	TP
Q	A	*	*	*	*	*	*	*	*	*	*	*	*	*	*	*	*	*	*	D	Q	*	*	*	*	*	*	*	*	*	*	TA
34	A	N	*	*	*	*	*	*	*	*	*	*	*	*	*	L	*	*	*	D	Q	*	*	*	*	*	*	*	*			TP
38	A	*	*	*	*	*	*	*	*	*	*	*	*	*	*	L	*	*	*	G	Q	*	*	*	*	*	*	S	*			TP
61	T	*	*	*	*	*	G	D	*	*	*	*	*	*	*	*	*	*	*	G	A	S	T	S	*	Q	L	G	–			TA
62	T	*	*	*	*	*	G	D	*	*	*	*	*	*	*	*	*	*	*	D	*	*	*	*	*	*	–	Q	L	G	*	TA
162-3	A	*	*	*	*	*	*	*	*	*	*	*	*	G	*	*	*	*	*	G	Q	*	*	*	*	*	*	*	*			TA
Ph21	A	*	*	*	*	*	G	D	*	*	*	*	*	*	*	*	*	*	*	G	A	S	T	S	*	Q	L	G				TA
Ch15	A	*	*	*	*	*	*	*	*	*	*	*	*	G	*	*	*	*	*	G	Q	*	*	*	*	*	*	*	*			TA

Asterisks indicate identical amino acids and gaps indicate deletions/insertions with respect to the reference repeat A. Amino acids at position 20 are in bold and underlined. Association of TR according to presence (TP) or absence (TA) of Rhipicephalus microplus on the farm.

1 The association was statistically significant by Chi square, Yates corrected test (p < 0.05).

Based on the data set provide by Repeat Analyzer we found 22 new strains. The strains τ-10-15 and α-β-β-β-Γ were most prevalent (Table 2). We found circulation of more than one genotype in ten *A. marginale* outbreaks (10/30).

**Table 2:** Characterization of *Anaplasma marginale* strains according to the combination of tandem repeat.

***N***	**Combination of TR**	**AA position 20^′^**								**Previously describe strains (countries/state)**
9	τ-10-15	G	G	G						Yes (Argentina/Chaco, Villa Angela; Brazil/ Parana, São Paulo, Lins; México/Nayarit, Santiago Ixcuintla)
6	α-β-β-β-Γ	G	G	G	G	D				Yes (Argentina/Santa Fe; México/Nayarit, Santiago Ixcuintla, Jalisco Tapalpa; Brazil/São Paulo, Goías, Mato Groso do Sul)
4	E-ru6-ru6	E	E	E						New
4	Γ-γ-9-3-5-9-3-15	D	D	D	G	D	D	G	G	New
3	EV7-10-15	G	G	G						New
3	τ-15-15	G	G	G						New
3	α-β-β-β-F	G	G	G	G	G				Yes (Brazil/ Rio de Janeiro)
3	F-β-β-Γ-γ	G	G	G	D	D				New
2	τ-154	G	D							New
2	E-ru6-ru6-ru6	E	E	E	E					New
2	T-B-B-B-M	D	D	D	D	G				Yes (Cuba/La Habana, Mayabeque)
2	3-3-ru6	G	G	E						New
2	4-15-15-15-15	G	G	G	G	G				New
1	Ph21-62-61	G	D	G						New
1	34-13-13-τ-38	D	D	D	G	G				New
1	B-Q-B-M-Q-B-M	D	D	D	G	D	D	G		New
1	162.3-Ch15-F-F-F-F	G	G	G	G	G	G			New
1	B-M	D	G							New
1	F-m-M-M-M-M	G	G	G	G	G	G			New
1	9-3-5-9-3-15	D	G	D	D	G	G			New
1	τ 15 10 15	G	G	G	G					New
1	F-F-F-4	G	G	G	G					New
1	3-F-100	G	G	G						New
1	F-F-100	G	G	G						New
1	F-F-F-F	G	G	G	G					New
1	B-B-B-C	D	D	D	G					Yes (USA/South Dakota Platte, Washington)
1	B-B-M	D	D	G						Yes (Argentina/Santa Fe, Pilar, Salta)
1	τ-15	G	G							New
60										

The amino acid at position 20′ of each TR is shown. New and previously described strains are shown. n, number of samples; TR, Tandem repeat; AA, amino acid.

In 46.7% (28/60) of our samples, the strains had the amino acid glycine (G) at position 20 of the MSP1a tandem repeat, 40% (24/60) had G and aspartate acid (D), 10% (6/60) had glutamic acid (E), and only 3.3% (2/60) had a mixture of E and G (Table 2). The data presented in the study are deposited in the Genbank repository, accession numbers OP382972-OP383031.

### Phylogenetic analysis

**Figure 2:**
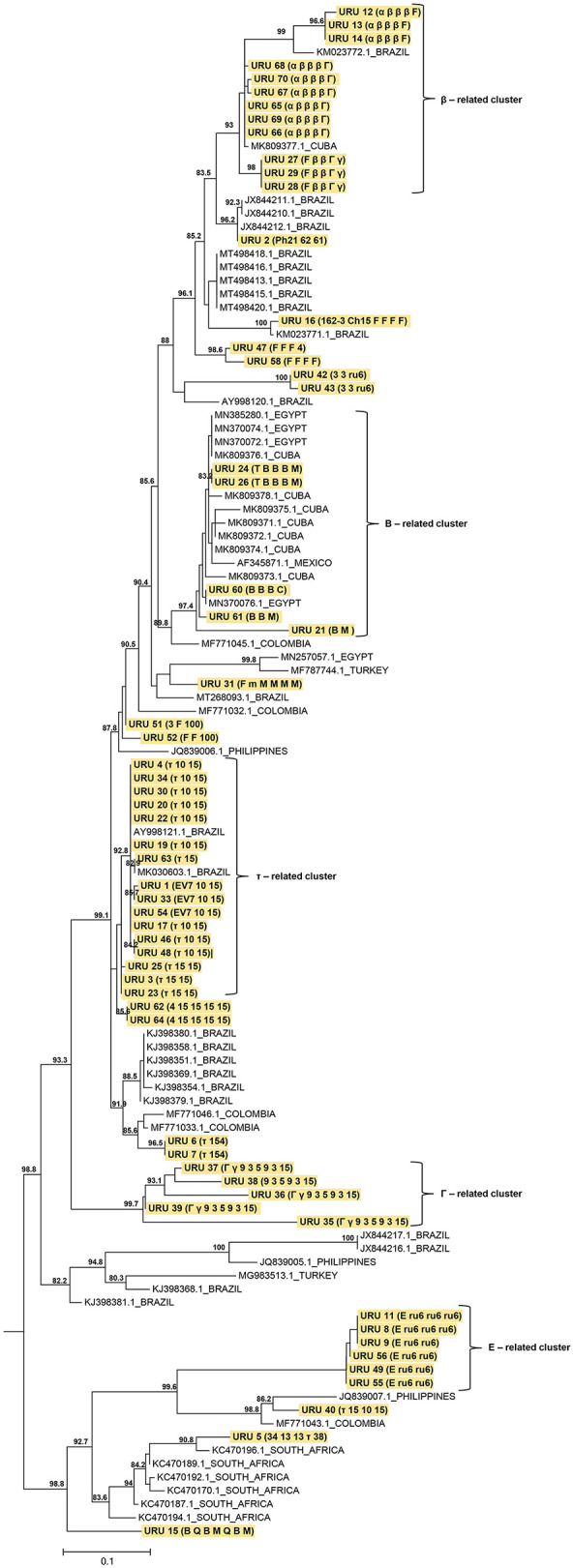
Maximum likelihood (ML) phylogenetic tree of *A. marginale* strains based in partial MSP1a amino acid sequences. The analysis involved 114 amino acid sequences (strains found in this study were highlighted in yellow). The ML phylogenetic tree was midpoint rooted and only SH-aLRT support values >80 are shown. The branch lengths are drawn to scale with the bar at the bottom indicating amino acid substitution per site. Combinations of tandem repeats are shown in parenthesis.

The results of phylogenetic analysis using MSP 1a amino acid sequences demonstrated the heterogeneity of the strains found in this study. Most of our sequences were closely related to sequences from Brazil. The phylogenetic tree showed a cluster that agreed with the structure of the tandem repeats. A large part of our sample was grouped into five clusters related to the tandem repeats β, B, τ, Γ, and E. Twelve, 5, 16, 5, and 6 samples were grouped based on the tandem repeats β, B, τ, Γ, and E, respectively, with 93, 97.4, 92.8, 99.7, and 100 support values (Figure 2).

### Statistical analysis

Statistical analysis of tandem repeats using phi correlation was performed considering the presence or absence of *R. microplus*. We classified the repeats into two classes: TR found in the presence (TP) or absence (TA) of ticks. Only the repeats Γ, β, α, and γ were found in the TA, with a statistically significant correlation (*p* < 0.05; Table 1).

## Discussions

Bovine anaplasmosis caused by *A. marginale* is one of the main parasitic diseases transmitted biologically or mechanically by vectors, which causes significant losses to the livestock industry. It is highly prevalent in tropical and subtropical regions (7, 27, 32, 33). As observed in this study, the bovine anaplasmosis mainly affects adult animals, which is consistent with several reports (1, 34, 35). In contrast, young cattle often evolve into subclinical diseases, responding more efficiently and developing a rapid immune response (36, 37).

Several studies worldwide have been conducted to characterize *A. marginale* strains, for which the major surface protein genes are widely used (15). MSP1a is one of the six MSP-reported proteins and has been described as a model molecule for the analysis of *A. marginale* genetic diversity (14, 26). This protein is involved in the adhesion of *A. marginale* to bovine erythrocytes and ticks gut cells. It plays an important role in cattle infection, tick transmission, and development of bovine immunity against *A. marginale* (3). The major surface protein 1a gene has a molecular size between 630 and 1,200 bp (24). This length polymorphism in MSP 1a is due to the variation in the number of TRs (12). The sizes of the sequenced samples were within this range.

During strain genotyping based on the microsatellite structure, we observed that genotype E was the most prevalent. These results are consistent with previous results for strains found mainly in Brazil (19, 20, 38), Argentina (18) and Colombia (39). Other genotypes frequently observed in our work were G and H. Studies carried out in Brazil (São Paulo and Maranhão) reported that genotype H was the most frequently found in beef and dairy cattle (21, 22). However, the genetic diversity of circulating strains in the equatorial zone, comprising the countries of Ecuador, Mexico, Cuba, and subtropical countries such as South Africa and the USA, are different, with G and C being the main genotypes (15, 27, 40).

The work carried out by Estrada-Peña et al., (14) reports that clusters of microsatellite-genotyped strains were observed according to the geographic region. Furthermore, the same author described that SD-ATG length (between 19 and 29 nucleotides) of the MSP1a microsatellite has been correlated with expression of the gene, which affects pathogen infection and transmission of *A. marginale*. The expression of MSP1a is lower in variants with an SD-ATG distance of 19 nucleotides, while those with a distance of 23 and 29 nucleotides have higher MSP1a gene expression (14). In our study, 88% (53/60) of the strains corresponded to genotypes with a distance of 23 nucleotides, suggesting that these *A. marginale* strains may have high infectivity.

In addition, in this study, based on the information provided by Repeat Analyzer 22 new strains were found. The τ-10-15 and α-β-β-β-Γ strain was overrepresented, which may be because we worked with clinical cases. It is possible that this strain is more pathogenic than the other strains reported. Theses strains have been previously described as the most prevalent strain in cattle in Brazil, Argentina and México (4, 20, 38). Correlation analysis of the tandem repeats, based on the presence or absence of a tick, revealed that only four repeats (Γ, β, α, and γ) were associated (*p* < 0.05) with the absence of *R. microplus*, which suggests that strains containing these repeats may be less adapted to tick transmission. Similar studies carried out in Argentina observed that repeat M was associated (*p* < 0.05) with the absence of *R. microplus* (18). Due to the high genetic diversity of the strains found in our region and the small number of samples analyzed, more studies must be carried out to validate the provisional results shown here.

The high genetic diversity of *A. marginale* in tick enzootic regions facilitated the co-circulation of several strains during the same outbreak. Argentinian studies have reported the presence of more than one *Anaplasma* strain infecting the same animal (18), as well as Barbosa et al. reported on work carried out in Angus cattle in Brazil (22).

Previous work carried out by de la Fuente et al. (41) demonstrated that tandem repeated peptides containing the amino acids D or E at position 20 of their tandem repeats have the ability to bind to tick cells. G-containing peptides do not bind to tick cell extracts. In this study, we observed that 53.3% of our samples contained D or E at position 20 of their tandem repeats, whereas 46.7% had G. These findings indicate that >50% of the strains that we characterized have the ability to bind to ticks cells and be biologically transmitted, but the remaining proportion does not. More studies are necessary to discover competent vectors of *A. marginale* strains that do not have the capacity to be biologically transmitted by ticks, as tabanids, *Stomoxys calcitrans* and other mechanical vector.

A phylogenetic tree revealed the co-circulation of different *A. marginale* strains in Uruguay. This provides valuable information on the distribution of strains, as reported in other studies (5, 14). A cluster related to TR was observed, in agreement with other reports (19, 42). Several of the strains found in this study are related to strains from Brazil. This could be attributed to Uruguayan cattle entering from southern Brazil and northern Argentina (43), bringing with them *R. microplus* ticks and different strains of *A. marginale*. These strains have to adapt to different factors such as climatic conditions, host breed, host immune response, population dynamics of ticks and insects that transmit them, transportation of cattle, and use of insecticides and acaricides (15, 41). This adaptation most likely caused the strains to evolve and generate a wide genetic diversity.

Further genetic studies based on the MSP1a protein are imperative, as it has been described as the reference gene for monitoring genetic diversity and evolution, an important identity marker of *Anaplasma marginale* strains. It also provides important information regarding the epidemiological behavior of these species. Finally, as it is an immunoreactive protein, the information provided here may be used for future studies on the design of vaccines for the control of this disease.

## Conclusion

This is the first study conducted in Uruguay to genetically characterize *A. marginale* strains. Analysis of MSP1a revealed the co-circulation of different strains identified in clinical diseases in cattle. This study provides valuable epidemiological information for understanding bovine anaplasmosis as well as basic information for the design of potential vaccines for the control and prevention of this disease.

## Data availability statement

The data presented in the study are deposited in the Genbank repository, accession numbers OP382972-OP383031.

## Ethics statement

This study did not require ethical approval because the samples that we used was provided by animals with natural infections that arrived at the laboratory of the Division of Veterinary Laboratories (DILAVE) of the Ministry of Livestock, Agriculture and Fisheries (MGAP) for diagnosis. It was a convenience sampling. Written informed consent was obtained from the owners for the participation of their animals in this study.

## Author contributions

PP, MA-F, RR, and JV contributed to conception, design of the study, and performed the experiments. MS provided the statistical analysis. PP, DM, MB-G, and NR-O characterization and analysis of genetic sequences. PP and JV conducted writing—reviewing. PP, RR, and JV acquired the funding. All authors contributed to the article and approved the submitted version.

## Funding

The research that gives rise to the results presented in this publication received funds from the Instituto Nacional de Investigación Agropecuaria (INIA), Uruguay, by the project CL_35: Determination of the current situation of *Rhipicephalus microplus* and tick fever and integrated control of both diseases, Animal Health Platform.

## Conflict of interest

The authors declare that the research was conducted in the absence of any commercial or financial relationships that could be construed as a potential conflict of interest.

## Publisher's note

All claims expressed in this article are solely those of the authors and do not necessarily represent those of their affiliated organizations, or those of the publisher, the editors and the reviewers. Any product that may be evaluated in this article, or claim that may be made by its manufacturer, is not guaranteed or endorsed by the publisher.
