# Supplementary material for: Characterization of strains of Anaplasma marginale from clinical cases in bovine using major surface protein 1a in Uruguay
Source: Front Vet Sci. 2022 Sep 20;9:990228. doi: 10.3389/fvets.2022.990228 (PMC9531168; doi:10.3389/fvets.2022.990228)
Supplement: Supplementary file 1 [file Table_1.docx]

**Supplementary Table SI.** Genetic characterization of *A. marginale* strains from 60 infected animal from 30 anaplasmosis outbreaks of Uruguay

| **Strains Id** | **GenBank** | **Department** | | **Production** | **Class** | **Obr.Id** | **MS** | **Tandem repeat** | **No. Of repeats** | **Tick** | **Previously described strain** |
| --- | --- | --- | --- | --- | --- | --- | --- | --- | --- | --- | --- |
| Uru1 | OP382972 | | Rio Negro | DC | Cow | 1 | E | EV7-10-15 | 3 | No | New |
| Uru2 | OP382973 | | Paysandú | BC | Cow | 2 | E | Ph21-62-61 | 3 | No | New |
| Uru3 | OP382974 | | Paysandú | BC | Cow | 2 | E | τ-15^2^ | 3 | No | New |
| Uru4 | OP382975 | | Rio Negro | BC | Cow | 3 | E | τ-10-15 | 3 | No | Yes |
| Uru5 | OP382976 | | Salto | BC | Steer | 4 | G | 34-13^2^- τ-38 | 5 | Yes | New |
| Uru6 | OP382977 | | Salto | BC | Steer | 4 | E | τ 154 | 2 | Yes | New |
| Uru7 | OP382978 | | Salto | BC | Steer | 4 | E | τ 154 | 2 | Yes | New |
| Uru8 | OP382979 | | Soriano | BC | Cow | 5 | H | E-ru6^3^ | 4 | No | New |
| Uru9 | OP382980 | | Salto | BC | Cow | 6 | H | E-ru6^2^ | 3 | Yes | New |
| Uru11 | OP382981 | | Salto | BC | Cow | 6 | H | E-ru6^3^ | 4 | Yes | New |
| Uru12 | OP382982 | | Paysandú | BC | Cow | 7 | E | α-β^3^-F | 5 | No | Yes |
| Uru13 | OP382983 | | Paysandú | BC | Cow | 7 | E | α-β^3^-F | 5 | No | Yes |
| Uru14 | OP382984 | | Paysandú | BC | Cow | 7 | E | α-β^3^-F | 5 | No | Yes |
| Uru15 | OP382985 | | Artigas | BC | Cow | 8 | G | B-Q-B-M-Q-B-M | 7 | No | New |
| Uru16 | OP382986 | | Artigas | BC | Cow | 8 | E | 162,3-Ch15-F^4^ | 6 | No | New |
| Uru17 | OP382987 | | Artigas | BC | Cow | 8 | E | τ-10-15 | 3 | No | Yes |
| Uru19 | OP382988 | | Paysandú | BC | Steer | 9 | E | τ-10-15 | 3 | Yes | Yes |
| Uru20 | OP382989 | | Paysandú | BC | Steer | 9 | E | τ-10-15 | 3 | Yes | Yes |
| Uru21 | OP382990 | | Paysandú | BC | Steer | 9 | E | B-M | 2 | Yes | New |
| Uru22 | OP382991 | | Paysandú | BC | Steer | 9 | E | τ-10-15 | 3 | Yes | Yes |
| Uru23 | OP382992 | | Paysandú | BC | Steer | 9 | E | τ-15^2^ | 3 | Yes | New |
| Uru24 | OP382993 | | Paysandú | BC | Steer | 10 | G | T-B^3-^M | 5 | Yes | Yes |
| Uru25 | OP382994 | | Paysandú | BC | Steer | 10 | E | τ-15^2^ | 3 | Yes | New |
| Uru26 | OP382995 | | Paysandú | BC | Steer | 10 | G | T-B^3^-M | 5 | Yes | Yes |
| Uru27 | OP382996 | | Rio Negro | BC | Cow | 11 | E | F-β^2^- Γ-γ | 5 | No | New |
| Uru28 | OP382997 | | Rio Negro | BC | Cow | 11 | E | F-β^2^- Γ-γ | 5 | No | New |
| Uru29 | OP382998 | | Rio Negro | BC | Cow | 11 | E | F-β^2^- Γ-γ | 5 | No | New |
| Uru30 | OP382999 | | Paysandú | BC | Steer | 12 | E | τ 10 15 | 3 | Yes | Yes |
| Uru31 | OP383000 | | Paysandú | BC | Heifer | 13 | E | F-m-M^4^ | 6 | Yes | New |
| Uru33 | OP383001 | | Paysandú | BC | Cow | 14 | E | EV7-10-15 | 3 | No | New |
| Uru34 | OP383002 | | Paysandú | BC | Cow | 15 | E | τ-10-15 | 3 | No | Yes |
| Uru35 | OP383003 | | Paysandú | BC | Cow | 16 | E | Γ-γ-9-3-5-9-3-15 | 8 | No | New |
| Uru36 | OP383004 | | Paysandú | BC | Cow | 16 | E | Γ-γ-9-3-5-9-3-15 | 8 | No | New |
| Uru37 | OP383005 | | Paysandú | BC | Cow | 16 | E | Γ-γ-9-3-5-9-3-15 | 8 | No | New |
| Uru38 | OP383006 | | Paysandú | BC | Cow | 16 | E | 9-3-5-9-3-15 | 6 | No | New |
| Uru39 | OP383007 | | Paysandú | BC | Cow | 16 | E | Γ-γ-9-3-5-9-3-15 | 8 | No | New |
| Uru40 | OP383008 | | Paysandú | DC | Cow | 17 | C | τ 15 10 15 | 4 | No | New |
| Uru42 | OP383009 | | Paysandú | BC | Cow | 18 | G | 3^2^- ru6 | 3 | Yes | New |
| Uru43 | OP383010 | | Paysandú | BC | Cow | 18 | G | 3^2^- ru6 | 3 | Yes | New |
| Uru46 | OP383011 | | Paysandú | BC | Cow | 19 | E | τ-10-15 | 3 | No | Yes |
| Uru47 | OP383012 | | Paysandú | BC | Cow | 19 | E | F^3^-4 | 4 | No | New |
| Uru48 | OP383013 | | Rio Negro | BC | Cow | 20 | E | τ-10-15 | 3 | Yes | Yes |
| Uru49 | OP383014 | | Rio Negro | BC | Cow | 21 | H | E-ru6^2^ | 3 | Yes | New |
| Uru51 | OP383015 | | Rio Negro | BC | Cow | 21 | E | 3-F-100 | 3 | Yes | New |
| Uru52 | OP383016 | | Paysandú | DC | Cow | 22 | E | F^2^-100 | 3 | No | New |
| Uru54 | OP383017 | | Paysandú | BC | Calf | 23 | E | EV7-10-15 | 3 | Yes | New |
| Uru55 | OP383018 | | Salto | BC | Cow | 24 | H | E-ru6^2^ | 3 | Yes | New |
| Uru56 | OP383019 | | Paysandú | BC | Cow | 25 | H | E-ru6^2^ | 3 | Yes | New |
| Uru58 | OP383020 | | Paysandú | BC | Steer | 26 | E | F^4^ | 4 | Yes | New |
| Uru60 | OP383021 | | Paysandú | BC | Cow | 27 | G | B^3^-C | 4 | Yes | Yes |
| Uru61 | OP383022 | | Paysandú | BC | Cow | 28 | G | B^2^-M | 3 | No | Yes |
| Uru62 | OP383023 | | Colonia | DC | Cow | 29 | E | 4- 15^4^ | 5 | No | New |
| Uru63 | OP383024 | | Colonia | DC | Cow | 29 | E | τ -15 | 2 | No | New |
| Uru64 | OP383025 | | Colonia | DC | Cow | 29 | E | 4- 15^4^ | 5 | No | New |
| Uru65 | OP383026 | | Colonia | DC | Cow | 30 | E | α-β^3^-Γ | 5 | No | Yes |
| Uru66 | OP383027 | | Colonia | DC | Cow | 30 | E | α-β^3^-Γ | 5 | No | Yes |
| Uru67 | OP383028 | | Colonia | DC | Cow | 30 | E | α-β^3^-Γ | 5 | No | Yes |
| Uru68 | OP383029 | | Colonia | DC | Cow | 30 | E | α-β^3^-Γ | 5 | No | Yes |
| Uru69 | OP383030 | | Colonia | DC | Cow | 30 | E | α-β^3^-Γ | 5 | No | Yes |
| Uru70 | OP383031 | | Colonia | DC | Cow | 30 | E | α-β^3^-Γ | 5 | No | Yes |
|  |  | |  |  |  |  |  |  |  |  |  |

Obr.Id= Outbreak Id, MS= Microsatellite; Production Beef Cattle =BC or Dairy Cattle =DC
